# Biomarkers in Alzheimer’s disease: Evaluation of platelets, hemoglobin and vitamin B12

**DOI:** 10.1590/1980-57642020dn14-010006

**Published:** 2020

**Authors:** Gustavo Alves Andrade dos Santos, Paulo Celso Pardi

**Affiliations:** 1 Universidade de São Paulo – USP. Faculdade de Medicina de Ribeirão Preto. Departamento de Anatomia e Cirurgia. Ribeirão Preto, SP, Brazil.; 2 Centro Universitário do Senac – Unidade Tiradentes. Departamento de Pós-graduação em Farmácia.; 3 Universidade Anhanguera Guarulhos. Departamento de Biomedicina, São Paulo, SP, Brazil.

**Keywords:** Alzheimer’s disease, biomarkers, dementia, vitamin B12, hemoglobin, platelets, doença de Alzheimer, biomarcadores, demência, vitamina B12, hemoglobina, plaquetas

## Abstract

Currently, the most likely hypotheses as the cause of Alzheimer’s disease are deposition of amyloid beta peptide in the cerebral cortex and hyperphosphorylation of Tau protein. The diagnosis of Alzheimer’s disease is based on the exclusion of other diseases, behavioral assessments, and blood and imaging tests. Biotechnology has created interesting perspectives for the early detection of Alzheimer’s disease through blood analysis, with special attention to platelets, hemoglobin and vitamin B12.

**Objective:**

To evaluate the concentrations of platelets, hemoglobin and vitamin B12 in the blood of older adults with and without dementia of Alzheimer’s disease.

**Methods:**

A case-control study involving 120 individuals was conducted, seeking to establish a correlation between changes in platelet, hemoglobin and vitamin B12 concentrations in patients with confirmed AD and in individuals in the inclusion group without AD. The study met the established ethical requirements.

**Results:**

Hemoglobin and platelet levels were statistically lower in patients with AD. The biochemical evaluation in AD patient and healthy groups for vitamin B12 showed a decrease in the levels of this compound in patients with AD.

**Conclusion:**

We demonstrated the feasibility of the use of blood biomarkers as predictive markers for the diagnosis of AD.

## RESUMO

Atualmente, as hipóteses mais prováveis como causa da doença de Alzheimer são a deposição do peptídeo beta amiloide no córtex cerebral e a hiperfosforilação da proteína Tau. O diagnóstico da doença de Alzheimer baseia-se na exclusão de outras doenças, avaliações comportamentais e exames de imagem e sangue. A biotecnologia criou perspectivas interessantes para a detecção precoce da doença de Alzheimer, pela análise sanguínea, com atenção especial às plaquetas, hemoglobina e vitamina B12.

**Objetivo:**

Avaliar as concentrações de plaquetas, hemoglobina e vitamina B12 no sangue de idosos com e sem demência de Alzheimer.

**Métodos:**

O estudo de caso-controle envolveu 120 indivíduos, buscando correlação entre mudanças nas concentrações de plaquetas, hemoglobina e vitamina B12 em pacientes com DA confirmada e indivíduos do grupo de inclusão, sem DA.

**Resultados:**

Os níveis de hemoglobina e plaquetas são estatisticamente mais baixos em pacientes com DA. A avaliação bioquímica em pacientes com DA e grupos saudáveis para vitamina B12 mostrou uma diminuição nos níveis deste composto em pacientes com DA.

**Conclusão:**

Demonstramos a viabilidade do uso de biomarcadores sanguíneos como marcadores preditivos para o diagnóstico de DA.

The search for a predictive diagnosis of Alzheimer’s disease is one of the biggest challenges for science. The diagnosis for Alzheimer’s disease is currently based on a full clinical evaluation, which includes behavioral and psychiatric assessment tests, as well as blood and imaging tests.^1^ AD was first presented by the German psychiatrist and neuropathologist Alois Alzheimer in November 1906 at the 37th Meeting of Psychiatrists in Southeast Germany, giving rise to one of the most important medical discoveries in the modern world. The case described as: “A disease peculiar to neurons of the cerebral cortex “ was his patient, Auguste D., 50 years old, who according to data collected from his clinical record, began to present cognitive deficits, loss of memory, confusion regarding time and space, with progressive worsening, leading to death five years later. Alois Alzheimer had identified necropsy in the brain tissue of this patient, the presence of distinct plaques and neurofibrillary tangles.^2^^-^ 5

Between 1906 and 1910, Emil Kraepelin, a psychiatrist also of German origin, named the new pathology as Alzheimer’s disease (AD) in honor of Alois Alzheimer. Thus, the term has been used for cases of this type of dementia, a condition which can also affect pre-senile patients, that is, before the age of 65.^6^

In the United States of America, estimates predict, that, by 2025, there will be 7.1 million AD patients aged 65 years or older, representing a 40% increase in the current five million Americans with AD.^7^

In Brazil, the rate of AD is estimated at 7.7 per 1000 people per year in individuals over 65 years. Every five years this rate practically doubles, with a higher incidence found among women, especially when older.^8^

Brooks and Bastouly (2004)^9^ argue that the diagnosis for AD can be defined based on clinical judgment, obtained from a thorough history and careful examination of mental state, where other causes leading to dementia should be excluded.

Besides evaluation using the MMSE (Mini-Mental State Examination), the authors cite:

- CSF test for the detection of amyloid peptide and Tau protein.
- Computed tomography.
- Electroencephalogram.
- Presence of the APOE4 allele.

Other pathologies should be excluded and some tests can support this conclusion:

- Complete blood count
- VHS
- Urea, creatinine
- Calcium
- Liver functions
- Serum levels of vitamin B12
- Folate
- Thyroid function
- Serology for syphilis
- Chest X-ray
- Computed tomography without contrast

In Brazil, Ordinance 1298 by the Ministry of Health (2013)^10^ established “Clinical Protocols and Therapeutic Guidelines” for AD and defines diagnostic criteria, inclusion and exclusion criteria, as well as treatments and regulation mechanisms.

The Brazilian consensus requires more examinations than the American equivalent, due to the profile of the Brazilian population and its miscegenation. The examination should be performed with the help of a family member or caregiver, aiming to identify memory, language and abilities, motor and visual coordination and abstract thoughts.^11^

The key challenge in the current clinical management of Alzheimer’s disease is the lack of an accurate biomarker for reliable diagnosis of the disease. The clinical features of AD overlap with a number of other dementia pathologies, and conclusive diagnosis can only be achieved at autopsy. A biomarker is objectively measured and evolved as an indicator of a normal biological process, pathogenic process or pharmacological response to a therapeutic intervention.^12^

When used in clinical trials, this marker may be defined as a laboratory measure that reflects the disease process activity.^12^^,^ 13

Many proteins have been measured in serum, plasma or platelets in a bid to find a peripheral marker for AD. The ideal biomarker for detecting AD must have specificity and sensitivity, as well as clinical diagnosis, being reliable and reproducible, easy to perform, low cost and noninvasive, such as blood, urine, saliva and busted scrapings studies. Invasive tests such as skin, rectal biopsies, bone marrow or cerebrospinal fluid (CSF) samples and even brain biopsy, are presented as drawbacks in clinical practice.^14^

In 2007, an International Working Group (IWG) proposed a new concept for AD through the discovery of biomarkers used to detect the disease. According to this group, a type of task force, AD is defined as a double clinical-biological entity that can be recognized *in vivo*, before the onset of dementia, by a hippocampal syndrome and evidence of biomarkers which may indicate the location or nature of AD and the changes caused by it.^15^

Considerable effort has been made to develop better diagnostic techniques for AD, which may pave the way for therapeutic efforts to be used increasingly early. Serum-based biomarkers may be the lowest cost and least invasive modality for routine screening and monitoring.

Anucleated platelets can be considered an available model to study the metabolic mechanisms that occur in the CNS, and related to AD. In addition, several intracellular signaling pathways important for platelet activation involve essential molecules, which have also been reported as modulating Amyloid Precursor protein (APP) processing.^16^ For decades, platelets have been considered an excellent model for studying neurodegenerative disorders, including AD.^17^

Platelets are one of the main elements involved in AD-associated vascular diseases such as stroke and atherosclerosis.^18^ Changes in blood flow induced by cerebral amyloid angiopathy or AD-related vascular diseases with consecutive occlusion-induced hypoperfusion of the vessels indicate another consequence of A*β* accumulation in the brain, besides its neurotoxicity.^19^ Platelets can be a good biomarker to investigate the onset of AD, where some studies have reported that platelets contain the amyloid protein precursor and secretase enzymes required for the amyloidogenic processing of this protein.^20^ Therefore, platelets not only reflect AD-related events in the brain, but may also influence AD progression. The molecular mechanisms involved and impact of platelets on AD are not well understood.^17^

Proteins, lipids and other metabolites can be examined in plasma, serum or cellular compartments. At these sites, erythrocytes, platelets or white cells may be identified and flow cytometry used for better verification. Cell studies can be obtained by culturing media for short periods and the respective results measured. Obviously, RNA can be obtained from cells, but is also present in plasma exosomes, an intriguing and potential source of biomarkers.^21^^-^ 23

Scientists acknowledge that low hemoglobin levels may be a kind of biomarker of ischemia associated with some events, such as cerebrovascular disease, changes associated with inducible factor hypoxia and also with hypoxia and erythropoietin levels, as well as changes associated with oxidative stress in heme regulation. Hemoglobin levels are associated with cognitive decline in domains other than episodic memory, thus suggesting a potential vascular cause. Low hemoglobin levels may be considered a predictive factor for the development of AD in older people.^24^ Erythropoietin stimulates erythropoiesis, reduces erythrosis and induces the formation of intracellular neural hemoglobins, which may exert beneficial effects regarding the onset and course of AD. There is evidence of a role of hemoglobin in the central nervous system as a possible candidate molecule involved in AD.^25^

There is an association between AD and up-regulation of proinflammatory cytokines, such as TNF-alpha specific genetic variants; IL-6; IFN-Gamma; and low plasma levels of vitamin B12.^26^

Vitamin B12 increases the concentrations of myelin metabolic markers and interferes with the integrity of plasmalogens, recognized as modulators of membrane dynamics. Plasmalogens are a specific type of phospholipid. In human health, the importance of plasmalogens is highlighted for their potential role in Alzheimer’s disease and other neurological disorders such as Down’s syndrome and Parkinson’s disease.^27^^-^ 28 Reduction in serum concentrations of Plasmalogens correlate with functional decline in patients with AD.^29^ Plasmalogens represent approximately 20% of the total phospholipid mass in humans and are widely distributed in tissues.^30^

In 2017, the Coalition Against Major Diseases (CAMD) conducted a large study on the use of algorithms in the diagnosis of AD. The substances evaluated were vitamin B12, serum sodium, liver enzymes, hemoglobin and cholesterol. The beneficial effects of vitamin B12 on cognition and a relationship between low hemoglobin corpuscular volume values and high Mini-Mental State Examination (MMSE) scores have been confirmed.^31^

The Alzheimer’s Association and the Alzheimer’s Drug Discovery Foundation in 2013 have universally selected top scientists to discuss the presence of biomarkers in the blood.^32^

## METHODS

This case control study was carried out in patients diagnosed with probable AD and cognitively healthy patients without AD at research centers in the cities of São Paulo and Cuiabá. The study was approved by the research ethics committee, under protocol CAAE 32791814.4.0000.5493. Hematological analyses were performed using a complete blood count and vitamin B12 levels. The collection, processing and analysis of the blood samples was done in accordance with the recommendations of the Brazilian Society of Clinical Pathology, and the tests were carried out at the Neolabor laboratory in São Paulo and the Clinical Laboratory of the Federal University of Mato Grosso in Cuiabá. All volunteers were advised to fast for the collection of samples (blood).

### Organization of groups

A total of 120 older adults were invited to participate in this experiment and were divided into two groups:

- Group without AD: 60 cognitively healthy individuals, with no diagnosis of AD, aged 60 years or older.
- Group with AD: 60 patients with diagnosis of probable AD.

The inclusion and exclusion criteria for classification of the volunteers without AD and the patients with AD were guided by Ordinance 1298 of 11/21/2013 by the Ministry of Health, which approves the clinical protocol and therapeutic guidelines of AD in Brazil.

All data from this experiment were statistically treated using GRAPH PAD PRISM 5.0 software. The non-parametric Mann-Whitney test was applied to compare the different groups (concentrations). The Kruskal-Wallis test was also applied for the comparison of experimental times. The level of significance of the null hypothesis was 5% (p ≤ 0.05).

## RESULTS

The data in Table 1 show the evaluation of hematological parameters of healthy patients and patients with AD during the experimental evaluation cycle performed in this study.

**Table 1:** Results of the hematological evaluation of the AD and No-AD groups (n = 60).

	AD	No-AD
Hemoglobin (g/dL)	12.87 ± 1.60	14.45 ± 0.87
Platelets (103/µL)	217.37 ± 49.49	228.75 ± 81.29

P < 0.001 increase in relation to Group AD (Kruskal-Wallis-Anova).

Hemoglobin and platelet levels were statistically lower in patients with AD. These data are consistent with the literature reporting that lower levels of hemoglobin are associated with cognitive impairment in AD.^33^ In addition, other factors related to the functioning of the hematologic system are also directly altered in patients with AD, such as homocysteine, vitamin B12 and folates. These findings reinforce the association of plasma homocysteine with cognitive impairment, although this is not exclusive to AD^33^ as these alterations may also be associated with depression.^34^

Table 2 shows the biochemical evaluation of the AD and healthy groups for Vitamin B12, revealing lower levels of this compound in patients with AD, as described by Faux.^33^

**Table 2:** Results of the values of the biochemical evaluation of the AD and healthy groups (n = 60).

	AD	No-AD
B12 Vitamin	267.72 ± 117.82	388.52 ± 58.68

P < 0.001 increase in relation to Group AD (Kruskal-Wallis-Anova).

## DISCUSSION

Recently, the association between serum platelet levels and the occurrence of AD has been suggested, confirming platelet dysfunctions in AD.^35^

Platelets are the first peripheral source of amyloid precursor protein (PPA). They have a proteolytic machinery capable of producing amyloid beta fragments (A*β*) similar to those produced in neurons. Platelets process PPA through the *α*-secretase pathway, releasing the soluble fraction of PPA (sPPA). Platelets produce small amounts of A*β*, more A*β*40than A*β*42. PPA and A*β* are stored in *α*-granules and released after platelet activation by thrombin and collagen, or agents that promote platelet degranulation.^36^

There have been reports of changes in platelet PPA expression,^36^ as well as alterations in serum platelet levels, in patients with AD.^37^

Through a known cerebral enzymatic pathway, platelets express the amyloid precursor protein (APP) and exhibit the complete mechanism for processing APP proteins into A*β* peptides.^16^

The search for precise diagnostic methods capable of predicting the onset of AD has been the subject of incessant research by scientists. Early identification of AD, through precise and efficient biomarkers, would promote a number of benefits, as shown in Table 1.

Identification of biomarkers is generally performed by blood or urine analysis, but with technological resources a large number of metabolites can be detected in urine. The new results obtained through research lend credence to the idea that the risk of AD can be determined early in people with mild cognitive disorders or even with normal aging.^38^

Platelets are known to play an important role in a variety of cardiovascular, psychosomatic, psychiatric, and neurodegenerative diseases. For this reason, platelets have been a promising target in the search for peripheral biomarkers in AD.^35^^,^ 39 Human platelets are known to be the source of more than 90% of circulating PPA protein^40^^-^ 42 and store A*β* in their granules, especially A*β*40, stimulated by physiological agonists such as thrombin, collagen or calcium.^40^^,^ 43 ^-^ 45

Investigations have been carried out into the expression of platelets in AD patients, showing alterations during some stages of the disease. The proportion of two isoforms, APP protein products that occur in platelets, was studied as a potential biomarker and was decreased in the platelet membranes of patients with AD and MCI when compared to control groups.^42^^,^ 46

Many studies have reported a significant decrease in platelet fractions in patients with AD, correlating them positively with cognitive decline.^46^^-^ 50

Previous studies have demonstrated the presence of hemoglobin in rodent and human neurons, thus indicating that hemoglobin is a normal component of nerve cells, and may play a role in intraneural oxygen homeostasis.^51^

A study of 5821 patients confirmed the feasibility of using biomarkers for the diagnosis of dementia, including hemoglobin and vitamin B12. Studies of more specific vitamin B12-related biomarkers, such as methylmalonic acid and holotranscobalamin, have associated mental decline with low B12 levels. In addition, hemoglobin levels, red blood cell counts and white blood cell counts are associated with low MMSE scores.^31^

This study sought to correlate the biomarker concentrations found in the blood of healthy volunteers and patients diagnosed with AD.

We conclude that Alzheimer’s disease throughout its evolution can lead to hematological alterations, especially in the levels of hemoglobin and platelets; in addition, reduced levels of vitamin B12 have been found. Further investigations are needed, involving the evaluation of substances in blood, such as platelets, vitamin B12 and hemoglobin, to prove the involvement of these components in patients with AD.

## References

[1] Alzheimer's Association (2015). 2015 Alzheimer's disease facts and figures. Alzheimers Dement.

[2] Maurer K, Volk S, Gerbaldo H (1997). Auguste D and Alzheimer's disease. Lancet.

[3] Maurer K, Maurer U (1998). Alzheimer: das leben eines Antes und die Karriere einer Krankheit.
Munich.

[4] Schachter AS, Davis KL (2000). Alzheimer's disease. Dialogues Clin Neurosci.

[5] Alzheimer A (1907). Über eine eigenartige Erkrankung der Hirnrinde. Allg Zschr Psychiatr Psych Gerichtl Med.

[6] Caramelli P, Viel AH (2006). 100 anos da doença de Alzheimer.

[7] Hebert LE, Weuve J, Scherr PA, Evans DA (2013). Alzheimer disease in the United States (2010-2050) estim ated
using the 2010 census. Neurology.

[8] Nitrini R, Caramelli P, Herrera E, Bahia VS, Caixeta LF, Radanovic M (2004). Incidence of dementia in a community-dwelling Brazilian
population. Alzheimer Dis Assoc Disord.

[9] Brooks BBJ, Bastouly V (2004). Doença de Alzheimer: uma visão histórica, genética, clínica e
terapêutica. Rev Medica na Costa.

[10] Ministério da Saúde (Brasil), Portaria nº 1.298, de 21 de novembro de 2013. (2013). Aprova o Protocolo Clínico e Diretrizes Terapêuticas da Doença de
Alzheimer.

[11] Aprahamian I, Martinelli J, Yassuda MS (2009). Doença de Alzheimer: revisão da epidemiologia e
diagnóstico. Rev Bras Clin Med.

[12] Blennow K, Hampel H, Weiner M, Zetterberg H (2010). Cerebrospinal fluid and plasma biomarkers in Alzheimer
disease. Nat Rev Neurol.

[13] Katz R (2004). Biomarkers and surrogate markers: an FDA
perspective. NeuroRx.

[14] Bermejo-Pareja F, Antequera D, Vargas T, Molina JA, Carro E (2010). Saliva levels of Abeta1-42 as potential biomarker of Alzheimer's
disease: a pilot study. BMC Neurol.

[15] Dubois B, Feldman HH, Jacova C, Hampel H, Molinuevo JL, Blennow K (2014). Advancing research diagnostic criteria for Alzheimer's disease:
the IWG-2 criteria. Lancet Neurol.

[16] Catricala S, Torti M, Ricevuti G (2012). Alzheimer disease and platelets: how's that
relevant. Immun Ageing.

[17] Gowert NS, Donner L, Chatterjee M, Eisele YS, Towhid ST, Münzer P (2014). Blood platelets in the progression of Alzheimer's
disease. PLoS One.

[18] Gawaz M, Langer H, May AE (2005). Platelets in inflammation and atherogenesis. J Clin Invest.

[19] Thal DR, Griffin WS, De Vos RA, Ghebremedhin E (2008). Cerebral amyloid angiopathy and its relationship to Alzheimer's
disease. Acta Neuropathol.

[20] Pluta R, Ulamek-Koziol M, Guest P (2019). Lymphocytes, Platelets, Erythrocytes, and Exosomes as Possible
Biomarkers for Alzheimer's Disease Clinical Diagnosis. Reviews on Biomarker Studies in Psychiatric and Neurodegenerative
Disorders. Advances in Experimental Medicine and Biology.

[21] Thambisetty M, Lovestone S (2010). Blood-based biomarkers of Alzheimer's disease: challenging but
feasible. Biomarkers Med.

[22] Hunter MP, Ismail N, Zhang X, Aguda BD, Lee EJ, Yu L (2008). Detection of microRNA expression in human peripheral blood
microvesicles. PLoS One.

[23] Simpson RJ, Lim JW, Moritz RL, Mathivanan S (2009). Exosomes: proteomic insights and diagnostic
potential. Expert Rev Proteomics.

[24] Shah RC, Buchman AS, Wilson RS, Leurgans SE, Bennett DA (2011). Hemoglobin level in older persons and incident Alzheimer disease:
prospective cohort analysis. Neurology.

[25] Altinoz MA, Guloksuz S, Schmidt-Kastner R, Kenis G, Ince B, Rutten BPF (2019). Involvement of hemoglobins in the pathophysiology of Alzheimer's
disease. Exp Gerontol.

[26] Politis A, Olgiati P, Malitas P, Albani D, Signorini A, Polito L (2010). Vitamin B12 levels in Alzheimer's disease: association with
clinical features and cytokine production. J Alzheimers Dis.

[27] Brites P, Waterham HR, Wanders RJ (2004). Functions and biosynthesis of plasmalogens in health and
disease. Biochim Biophys Acta.

[28] Nagan N, Zoeller RA (2001). Plasmalogens: biosynthesis and functions. Prog Lipid Res.

[29] Wood PL, Mankidy R, Ritchie S, Heath D, Wood JA, Flax J (2010). Circulating plasmalogen levels and Alzheimer Disease Assessment
Scale-Cognitive scores in Alzheimer patients Psychiatry. Neurosci.

[30] Braverman NE, Moser AB (2012). Functions of plasmalogen lipids in health and
disease. Biochim Biophys Acta.

[31] Szalkal B, Grolmusz VK, Grolmusz VI (2017). Identifying combinatorial biomarkers by association rule mining
in the CAMD Alzheimer's database. Arch Gerontology Geriatrics.

[32] Jack CR, Knopman DS, Jagust WJ, Petersen RC, Weiner MW, Aisen PS (2013). Tracking pathophysiological processes in Alzheimer's disease: an
updated hypothetical model of dynamic biomarkers. Lancet Neurol.

[33] Faux NG, Ellis KA, Porter L, Fowler CJ, Laws SM, Martins RN (2011). Homocysteine, vitamin B12, and folic acid levels in Alzheimer's
disease, mild cognitive impairment, and healthy elderly: baseline
characteristics in subjects of the Australian Imaging Biomarker Lifestyle
study. J Alzheimers Dis.

[34] Coppen A, Bolander-Gouaille C (2005). Treatment of depression: time to consider folic acid and vitamin
B12. J Psychopharmacol.

[35] Plagg B, Marksteiner J, Kniewallner KM, Humpel C (2015). Platelet dysfunction in hypercholesterolemia mice, two
Alzheimer's disease mouse models and in human patients with Alzheimer's
disease. Biogerontology.

[36] Evin G, Li QX (2012). Platelets and Alzheimer's disease: potential of APP as a
biomarker. World J Psychiatry.

[37] Veitinger M, Varga B, Guterres SB, Zellner M (2014). Platelets, a reliable source for peripheral Alzheimer's disease
biomarkers. Acta Neuropathol Commun.

[38] Sapkota S, Tran T, Huan T, Lechelt K, Macdonald S, Camicioli R (2015). Metabolomics analyses of salivar samples discriminate normal
aging; mild cognitive impairment; and Alzheimer's disease groups and produce
biomarkers predictive of neurocognitive performance. Alzheimers Dement.

[39] El Haouari M, Rosado JA (2009). Platelet function in hypertension. Blood Cells Mol Dis.

[40] Li QX, Berndt MC, Bush AI, Rumble B, Mackenzie I, Friedhuber A (1994). Membrane-associated forms of the beta A4 amyloid protein
precursor of Alzheimer's disease in human platelet and brain: surface
expression on the activated human platelet. Blood.

[41] Li QX, Fuller SJ, Beyreuther K, Masters CL (1999). The amyloid precursor protein of Alzheimer disease in human brain
and blood. J Leukoc Biol.

[42] Padovani A, Borroni B, Colciaghi F, Pettenati C, Cottini E, Agosti C (2002). Abnormalities in the pattern of platelet amyloid precursor
protein forms in patients with mild cognitive impairment and Alzheimer
disease. Arch Neurol.

[43] Bush AI, Martins RN, Rumble B, Moir R, Fuller S, Milward E (1990). The amyloid precursor protein of Alzheimer's disease is released
by human platelets. J Biol Chem.

[44] Kokjohn TA, Van Vickle GD, Maarouf CL, Kalback WM, Hunter JM, Daugs ID (2011). Chemical characterization of pro-inflammatory amyloid-beta
peptides in human atherosclerotic lesions and platelets. Biochim Biophys Acta.

[45] Skovronsky DM, Lee VM, Praticò D (2001). Amyloid precursor protein and amyloid beta peptide in human
platelets. Role of cyclooxygenase and protein kinase C. J Biol Chem.

[46] Borroni B, Agosti C, Marcello E, Di Luca M, Padovani A (2010). Blood cell markers in Alzheimer Disease: amyloid Precursor
Protein form ratio in plateletse. Exp Gerontol.

[47] Di Luca M, Pastorino L, Bianchetti A, Perez J, Vignolo LA, Lenzi GL (1998). Differential level of platelet amyloid beta precursor protein
isoforms: an early marker for Alzheimer disease. Arch Neurol.

[48] Di Luca M, Pastorino L, Cattabeni F, Zanardi R, Scarone S, Racagni G (1996). Abnormal pattern of platelet APP isoforms in Alzheimer disease
and Down syndrome. Arch Neurol.

[49] Tang K, Hynan LS, Baskin F, Rosenberg RN (2006). Platelet amyloid precursor protein processing: a biomarker for
Alzheimer's disease. J Neurol Sci.

[50] Zainaghi IA, Talib LL, Diniz BS, Gattaz WF, Forlenza OV (2012). Reduced platelet amyloid precursor protein ratio (APP ratio)
predicts conversion from mild cognitive impairment to Alzheimer's
disease. J Neural Transm (Vienna).

[51] Ferrer I, Gómez A, Carmona M, Huesa G, Porta S, Riera-Codina M (2011). Neuronal hemoglobin is reduced in Alzheimer's disease,
argyrophilic grain disease, Parkinson's disease, and dementia with Lewy
bodies. J Alzheimers Dis.

